# 

**Published:** 1975

**Authors:** 


### Contents

Gastric Leukoplakia Associated with Fundal Leiomyoma ... ... ... ... ... 45

Obituary—Arthur Wilfrid Adams ... ... ... 49

The Barber Surgeons of Bristol ... ... ... 51

Haemorrhage Complicating Anticoagulant Therapy ... ... ... ... ... ... 57

Final Examination for the Degrees of M.B., Ch.B. 61

Printed by F. Bailey & Son Ltd., Dursley, Glos.

2488

